# Pu-erh Tea Protects the Nervous System by Inhibiting the Expression of Metabotropic Glutamate Receptor 5

**DOI:** 10.1007/s12035-016-0064-3

**Published:** 2016-08-30

**Authors:** Chunjie Li, Shaomeng Chai, Yongzhi Ju, Lu Hou, Hang Zhao, Wei Ma, Tian Li, Jun Sheng, Wei Shi

**Affiliations:** 1 Key Laboratory for Molecular Enzymology and Engineering, Ministry of Education, Jilin University, Changchun, 130012 China; 2 College of Life Science, Jilin University, No. 2699 Qianjin Street, Jilin Province. Rm. 226 Life Science Building, Changchun, 130012 China; 3 Yunnan Research Centre for Advanced Tea Processing, Yunnan Agricultural University, Kunming, 650201 China; 4 Internal Medicine-Cardiovascular Department in China-Japan Union Hospital, Jilin University, Changchun, 130012 China; 5 School of Science and Technology in Changchun University of Science and Technology, Changchun, 130012 China

**Keywords:** Pu-erh tea, Glutamate, mGluR5, Neurodegenerative diseases

## Abstract

Glutamate is one of the major excitatory neurotransmitters of the CNS and is essential for numerous key neuronal functions. However, excess glutamate causes massive neuronal death and brain damage owing to excitotoxicity via the glutamate receptors. Metabotropic glutamate receptor 5 (mGluR5) is one of the glutamate receptors and represents a promising target for studying neuroprotective agents of potential application in neurodegenerative diseases. Pu-erh tea, a fermented tea, mainly produced in Yunnan province, China, has beneficial effects, including the accommodation of the CNS. In this study, pu-erh tea markedly decreased the transcription and translation of mGluR5 compared to those by black and green teas. Pu-erh tea also inhibited the expression of Homer, one of the synaptic scaffolding proteins binding to mGluR5. Pu-erh tea protected neural cells from necrosis via blocked Ca^2+^ influx and inhibited protein kinase C (PKC) activation induced by excess glutamate. Pu-erh tea relieved rat epilepsy induced by LiCl-pilocarpine in behavioural and physiological assays. Pu-erh tea also decreased the expression of mGluR5 in the hippocampus. These results show that the inhibition of mGluR5 plays a role in protecting neural cells from glutamate. The results also indicate that pu-erh tea contains biological compounds binding transcription factors and inhibiting the expression of mGluR5 and identify pu-erh tea as a novel natural neuroprotective agent.

## Introduction

**Fig. 1:**
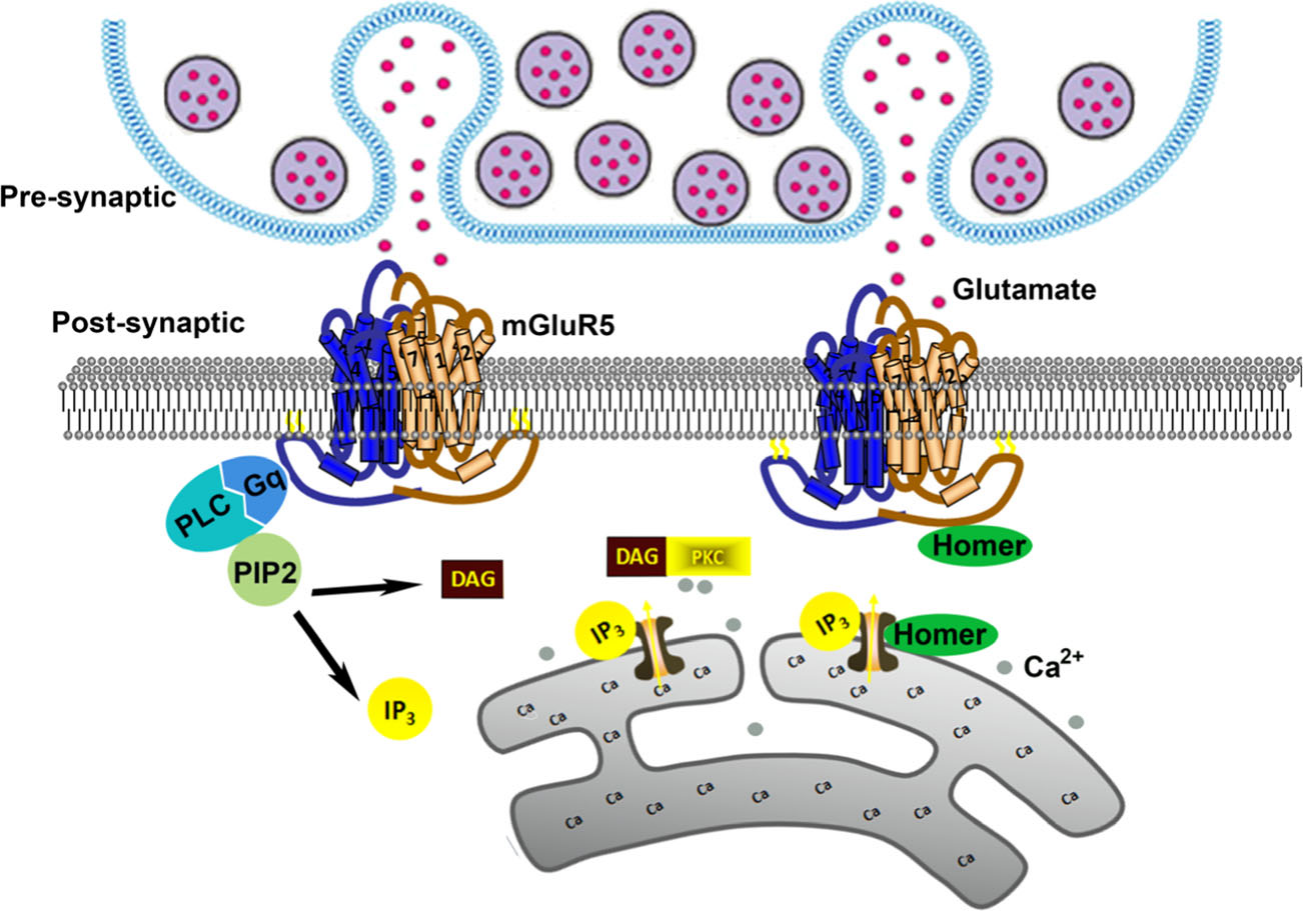
Schematic of mGluR5 signalling pathway

Glutamate, the primary excitatory neurotransmitter in the brain, plays various physiological roles in cognition, memory and perception via the activation of multiple receptors [1]. An increase in the extracellular levels of glutamate leads to disorganised neuronal spike activity, impairing the processing of relevant information in the brain [2]. Glutamate binding metabotropic glutamate receptors (mGluRs) are one of G-protein-coupled receptors and exert its physiological roles through intracellular chemical-messenger signalling cascades [3]. Eight mGluRs (mGluR1–8) have been discovered and are grouped into three categories: group I (mGluR1 and 5), group II (mGluR2 and 3) and group III (mGluR4, 6, 7 and 8) according to sequence identity, pharmacological properties and downstream activation [4]. In general, mGluR5 combined with glutamate activates the G-protein Gq/11, and subsequently the activated G-protein activates phospholipase C (PLC) which hydrolyses membrane phosphoinositides to form inositol 1, 4, 5-triphosphate (IP3) and diacylglycerol (DAG). IP3 causes the release of intracellular calcium, which activates PKC [5]. The Homer family binds to the intracellular C-terminal tail of mGluR5 and also excites downstream effectors (Fig. 1) [6]. It is noteworthy that mGluR5 regulates various mechanisms implicated in neurogenesis and synaptic maintenance, and the abnormal regulation of mGluR5 has been implicated in the pathophysiology of many neurological and psychiatric disorders, including pain, epilepsy, schizophrenia, drug addiction and Alzheimer’s disease [7]. Thus, mGluR5 represents a pathophysiological hallmark for neuroprotective agents of potential application in neurodegenerative disorders [8].

Epilepsy is the second most common neurological disorder after stroke and a major burden for public health systems, afflicting worldwide approximately about 65 million people [9]. Although current antiepileptic drugs have achieved complete seizure control, they do not prevent or eliminate the substantial behavioural, cognitive and somatic comorbidities in many epilepsy patients [10]. New drugs with fewer side reactions and greater efficacy are urgently needed. Epilepsy researchers have recently embarked on a revitalise effort to move from targeting the control of symptoms to strategies of prevention and cure. One important direction for new therapeutics is identifying new molecular targets [11].

Tea is one of the most popular beverages in the world owing to its refreshing taste and attractive aroma. Its possible beneficial health functions have recently received much research attention. Pu-erh tea, a fermented tea, produced mainly in Yunnan province of China, is obtained by first drying crude green tea leaves (*Camellia sinensis var. assamica* (L.) O. Kuntze; Theaceae) and then subjecting them to secondary fermentation by microorganisms, such as *Aspergillus* sp., resulting in a unique type of tea [12]. Several studies have reported that pu-erh tea has shown a wide range of biological effects, such as inhibiting nitric oxide production [13], promoting weight loss [14], reducing blood lipids [15], exerting antimicrobial and antitumor activities [16, 17] and scavenging free radicals [18]. We recently also found that pu-erh tea may reduce the incidence of epilepsy and amyotrophic lateral sclerosis [19, 20]. These results suggest that the potential efficacy of pu-erh tea in the regulation of the nervous system.

In this study, we discovered that pu-erh tea downregulated the transcription and translation of mGluR5 by Affymetrix Expression microarrays experiments and Western blot. Pu-erh tea also protected SH-SY5Y cells against apoptosis induced by L-glutamate (L-Glu) and alleviated epilepsy behaviour by inhibiting the expression of mGluR5. The results identified mGluR5 as a target for the treatment of neurological and psychiatric disorders and suggested that pu-erh tea might be a potential natural source of protection against neurodegenerative diseases associated with mGluR5. Accordingly, the aim of this study was to identify the protective mechanisms of pu-erh tea against injury to neuronal cells in vitro and in vivo.

## Materials and Methods

### Materials

Adult male Wistar rats (4–5 weeks old) were obtained from the Animal Resource Center of Jilin University and housed at 22 ± 2 °C in a controlled environment with a 12-h light and 12-h dark cycle. All rats were treated humanely and in compliance with the recommendations of the animal care committee of Jilin University. We used the minimum number of mice required to draw a conclusion and tried to minimise their suffering as much as possible.

3-(4, 5- Dimethylthiazol-2-yl)-2, 5-diphenyltetrazolium bromide (MTT), and dimethyl sulfoxide (DMSO), L-Glu were purchased from Sigma. Cell culture media, Dulbecco’s modified Eagle’s medium (DMEM), heat-inactivated foetal bovine serum (FBS) and trypsin were supplied by Gibco. Antibodies were purchased from Santa Cruz (CA93446, USA). A Luciferase Reporter Gene Assay Kit was purchased from Beyotime. (RS)-2-Chloro-5-hydroxyphenylglycine (CHPG), a selective mGluR5 agonist and 2-Methyl-6-(phenylethynyl)-pyridine (MPEP), a selective mGluR5 antagonist, were purchased from Selleckchem.

### Cell Culture

The SH-SY5Y, HEK293T and 3 T3 cell lines were cultured in DMEM, and the Jurkat cell line was cultured in RPMI1640 medium supplemented with 10 % FBS, 100 units/mL penicillin and 100 units/mL streptomycin (Invitrogen).

### Cytotoxicity Assay

In the cytotoxicity assay, cells were seeded at a density of 1 × 10^4^ cells/well in 96-well plates and incubated for 24 h. Cells were treated with pu-erh tea at various concentrations for 24 h. MTT (5 mg/mL, 20 μL) was added to the medium 24 h later. After incubation at 37 °C for 4 h, the supernatant was aspirated and 100 μL of DMSO was added to each well, followed by vibration for 10 min. The absorbance in the experimental wells was measured at 570 nm with a microplate reader.

### Affymetrix Microarrays

RNA was extracted with TRI reagent (Sigma) and DNAse I (Invitrogen) from Jurkat in triplicate with or without pu-erh tea treatment for 12 h. RNA quality and concentration were assessed with an Agilent 2100 Bioanalyser and Nanodrop ND-1000. Data from Affymetrix 430 2.0 microarray chips (SCIBLU, Affymetrix) was analysed with Arraystar 3 software (DNA STAR Inc.), which was quantile-normalised and processed by the RMA (Affymetrix) algorithm.

### Luciferase Assay

**Fig. 2:**
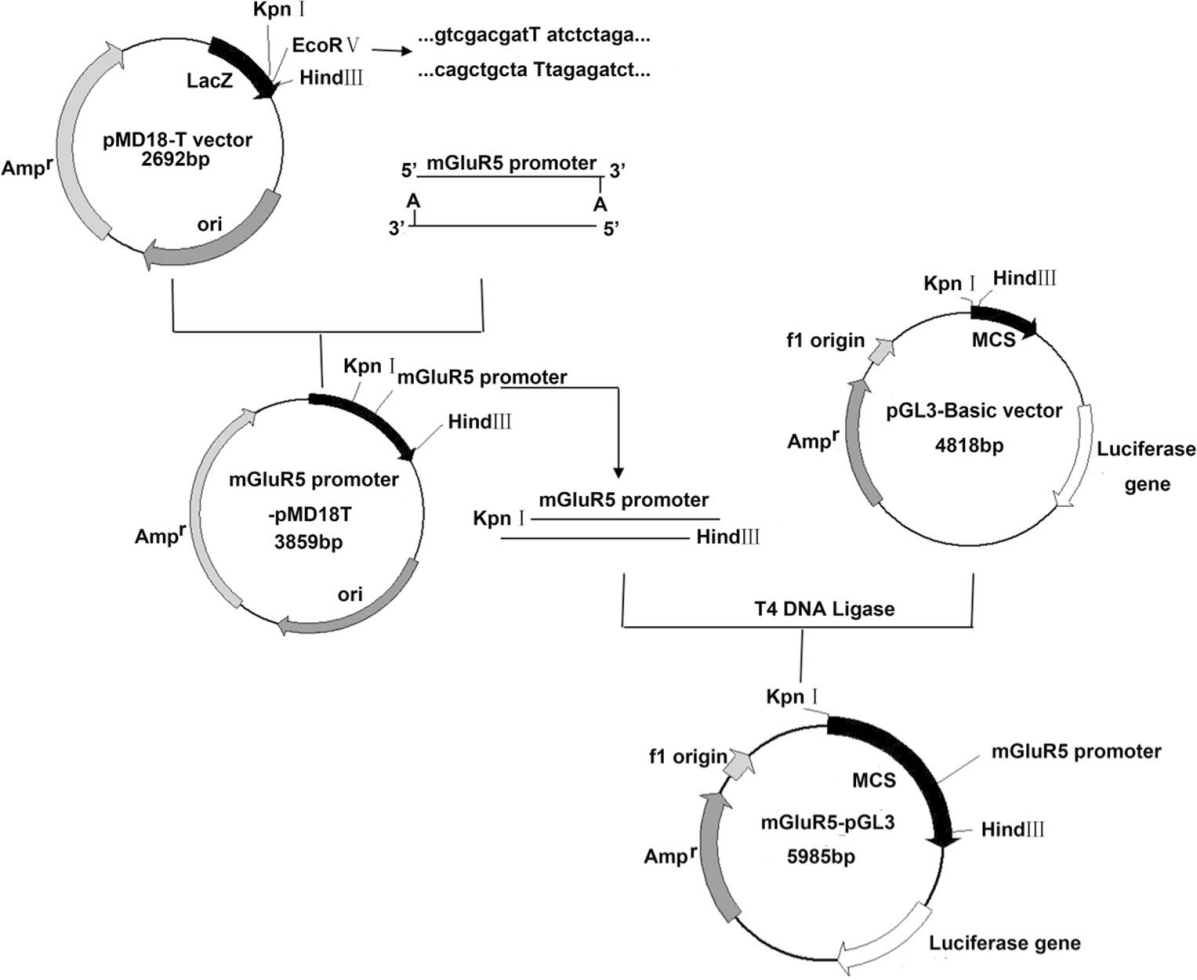
Construction strategy and structure of the recombinant plasmid mGluR5 promoter-pGL3

Promoter regions were amplified by PCR amplification and subcloned into the KpnI/HindIII sites of the luciferase reporter vector pGL3-Basic (Promega) upstream of the firefly luciferase gene (Fig. 2). SH-SY5Y and HEK293T cells were transfected with mGluR5 promoter-pGL3 recombinant plasmid by electroporation according to the manufacturer’s instructions. A monolayer of 80 % confluent cells was trypsinised, washed with appropriate media and washed again in ice-cold PBS buffer. Cell suspension for the electroporation was first prepared in 400 μL ice-cold PBS buffer (2.5 × 10^6^ cells/mL) in a 0.4-cm electric rotary cup, followed by adding 10 μg plasmid. The electroporation conditions were adopted from previous work, as follows: U = 225 V, R = ∞, Ω = 1050 μF. After electroporation, cells were seeded in 12-well plates at a density of 4 × 10^4^ cells per well. After 12 h, transfected cells were incubated with pu-erh, black and green teas. Cells were then harvested and firefly luciferase activity was determined using a Sirius luminometer (Berthold Detection System). Experiments were performed in triplicates and repeated at least three times independently.

### Western Blot

Cells were prepared in ice-cold lysis buffer (100 mM NaCl, 50 mM Tris-HCl, 1 mM EDTA, 10 mM MgCl_2_, pH 7.2) containing 1 % Triton X-100, phosphatase and protease inhibitor cocktail (Dinggguo, China) for 10 min. Cell lysates were centrifuged at 12,000*g* for 10 min at 4 °C, and protein concentrations of supernatants were determined with BCA assay. Lysates were added 5 × SDS containing 100 mM DTT. Equal amounts of protein were loaded onto and separated on 12 % SDS-PAGE gels and transferred to polyvinylidene fluoride (PVDF) membranes. Membranes were incubated with primary antibodies overnight, washed three times in washing buffer (0.1 % Tween-20 in PBS), incubated with HRP-coupled secondary antibodies for 1 h, washed three times in washing buffer and developed using chemiluminescent HRP detection substrate. When proteins were extracted from hippocampus tissue, hippocampus tissues were placed in lysis buffer (as above) and homogenised for 1 min. The homogenate was then centrifuged at 12,000*g* for 10 min at 4 °C. The final supernatant was collected and processed as above.

### Nuclear Staining with Hoechst 33342

SH-SY5Y cells were seeded in 6-well plates (1.5 × 10^5^ cells/well) for 24 h. Cells in a 6-well plate were incubated with pu-erh, black or green teas for 12 h before treatment with L-Glu for 24 h. Cells were washed with PBS and stained with 500 μL of Hoechst 33342 solution for 15 min at room temperature in the dark. The nuclear morphology of the cells was examined under a fluorescent microscope.

### Flow Cytometry Analysis of Apoptosis in SH-SY5Y

SH-SY5Y cells were seeded in 6-well plates (1.5 × 10^5^ cells/well) for 24 h. Cells in each 6-well plate were incubated with pu-erh, black or green teas for 12 h before treatment with L-Glu for 24 h. Cells treated with medium alone were used negative control. Apoptosis of cells was evaluated by measurement of the exposure of phosphatidylserine on the cell membranes using Apoptosis Detection Kits. Cell pellets were resuspended in a staining solution containing propidium iodide (PI) for 5 min and Annexin V-FITC for 15 min at room temperature in the dark. The cells were assessed by fluorescence-activated cell sorting (FACS) using the Cell Quest software (BD, Pharmingen).

### Calcium Imaging

SH-SY5Y cells were seeded in 6-well plates (1.5 × 10^5^ cells/well) for 24 h. Cells in a 6-well plate were incubated with pu-erh, black, or green teas for 12 h before treatment with L-Glu for 24 h. Cells treated with medium alone were used as negative control. Cells were loaded with 2 μM Fluo-3 AM at 37 °C for 1 h. Cells were suspended in PBS after being washed three times and analysed by a flow cytometry device equipped with the Cell Quest software at 488 nm.

### Epileptic Rat Model

Status epilepticus (SE) was induced in adult Wistar rats by using Licl-pilocarpin [21]. Rats were randomly divided into 6 groups with 6 rats in each group. Three groups were administered intragastricly with pu-erh tea at a dose of 0.5 g/Kg/day, the other three groups were treated with saline. After 20 days, lithium chloride was intraperitoneally injected into the four groups rats that two of them were dministered intragastricly with pu-erh tea, the other two were treated with saline at a 127 mg/kg dosage. After 18 h, the rats were treated with a 40 mg/kg dose of pilocarpine, administered intraperitoneally. Two of the groups that one was treated with pu-erh tea and Licl-pilocarpin, the other was treated with saline and Licl-pilocarpin were treated with 4 mg/kg diazepam, before pilocaroine administered intragastricly. Saline was administered to the control group at the same dosage.

### Behavioural Study

Rats were video monitored from day 0 to day 15 post status epilepsy (SE) and were scored by an individual blind to the experimental design. Behavioural seizure was recorded according to a modification of the classification of Racine [22]: 0, exploring; 1, immobility; 2, rigid posture; 3, head nodding; 4, bilateral forelimb clonus and falling; 5, continued clonus and falling; and 6, generalised tonus. Three behavioural patterns of SE could be recognised: I, initial (class 1–2), M, middle (class 3) and C, critical (class 4–6). Seizure number and type were recorded within 1 h of the seizure in day 0. Then, seizure number and type were recorded 30 min every 24 h from day 1 to day 15.

### Measurements of IP3 and DAG

The measurement of the contents of IP3 and DAG was conducted using IP3 and DAG ELISA kits (RD, USA). Hippocampus tissues were placed in ice-cold PBS and homogenised for 1 min. The homogenate was then centrifuged at 12,000*g* for 10 min at 4 °C. The final supernatant was collected.

### Haematoxylin and Eosin (HE) Staining

Brains were prepared from control and post-SE rats. Animals were anaesthetised using 20 % urethane at a dose of 0.5 mL/kg. Once deeply anesthetised, animals underwent heart perfusion with ice-cold modified PBS followed by 4 % poly-formaldehyde. The brain was removed and dehydrated in 20 and 30 % sucrose. The brains were then embedded in optimal cutting temperature compound (OCT). Six-micrometre-thick slices were made with a tissue slicer and stained in HE solutions to examine morphological changes.

### Immunofluorescence

To detect the expression of mGluR5 in rat brains, 6-μm paraffin sections were subjected to antigen retrieval and blocking of endogenous peroxidase activity, followed by incubation with mGluR5 monoclonal antibody (diluted at 1:200) overnight at 4 °C. The sections were incubated with FITC-conjugated anti-mouse IgG (1:250) for 2 h at room temperature. All images were acquired with a fluorescence microscope.

### Statistical Analysis

All values were expressed as mean ± S.D. A One-way analysis of variance (ANOVA) was used to detect statistical significance followed by post hoc multiple comparisons (Dunn’s test) by GraphPad Prism 6.0. A value of *P* < 0.05 was considered to be significant [23, 24].

## Results

### Pu-erh Tea Down-Regulated the Transcription of mGluR5

**Fig. 3:**
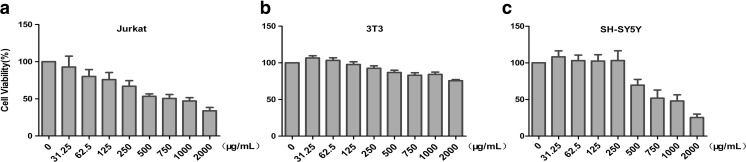
**a** Jurkat cell viability after incubation with various concentration of pu-erh tea for 24 h. **b** 3 T3 cell viability after incubation with various concentration of pu-erh tea for 24 h. **c** SH-SY5Y cell viability after incubation with various concentration of pu-erh tea for 24 h. Data were analysed using the GraphPad Prism software (*n* = 6)

To investigate the cytotoxicity of pu-erh tea, cell viability assays were performed. Our finding demonstrated that pu-erh did not inhibit the proliferation of Jurkat or SH-SY5Y at concentrations lower than 250 μg/mL, whereas it exerted a marked inhibitory effect on the proliferation of Jurkat and SH-SY5Y at concentrations greater than 0.5 mg/mL. No inhibition was observed in 3 T3 (Fig. 3).

The messenger RNA (mRNA) expression differences between the Jurkat cells treated and untreated with pu-erh tea were detected with Affymetrix expression microarrays. The results showed that pu-erh tea could downregulate mGluR5, one of the G protein-coupled receptors implicated in the nervous system (Tables 1, 2).

**Table 1:** Pu-erh tea extract increased the expression of target genes

UniGene.ID	Fold change	Gene. title
Hs.520028	14.54148	Heat shock 70 kDa protein 1 A
Hs.654614	10.09114	Heat shock 70 kDa protein 6
Hs.591670	6.948213	Inhibitor of DNA binding 2
Hs.658815	6.465138	Transcribed locus
Hs.525704	5.117358	Jun oncogene
Hs.155569	4.549088	ChaC
Hs.370699	3.700921	Hypothetical protein LOC284801

**Table 2:** Pu-erh tea extract decreased the expression of the target gene

UniGene.ID	Fold change	Gene. title
Hs.188859	−1.705	G protein-coupled receptor 20
Hs.446091	−1.705	Wilms tumour 1 associated protein
Hs.631992	−1.717	Dystonin
Hs.122125	−1.719	Transcribed locus
Hs.697691	−1.77	Olfactory receptor
Hs.370036	−1.805	Chemokine (C-C motif) receptor 7
Hs.135232	−1.817	Transcribed locus
Hs.147361	−1.936	Metabotropic glutamate receptor 5
Hs.224012	−1.995	Jagged 1 (Alagille syndrome)
Hs.5462	−2.017	Solute carrier family 4
Hs.1466	−2.039	Glycerol kinase
Hs.660607	−2.073	Oestrogen receptor 2 (ER beta)
Hs.76884	−2.317	Inhibitor of DNA binding 3
Hs.504609	−4.464	Inhibitor of DNA binding 1

### Pu-erh Tea Inhibited the Activity of mGluR5 Promoter

**Fig. 4:**
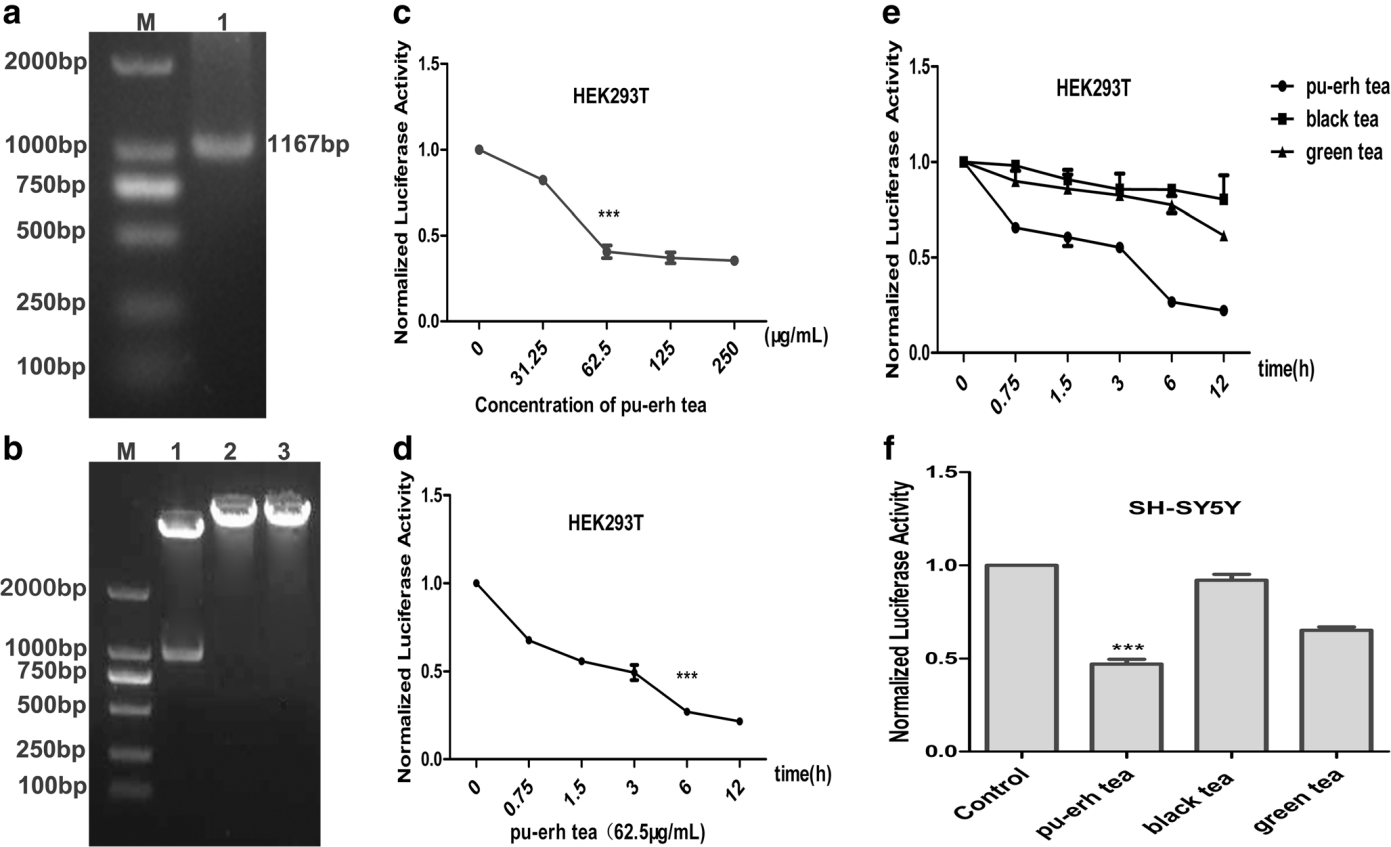
**a** Agarose gel electrophoresis of mGluR5 promoter. M: 2000 DNA marker; 1:mGluR5 promoter PCR product. **b** Agarose gel electrophoresis of mGluR5 promoter-pGL3 double restriction enzyme digestion. M: 2000 bp; 1: mGluR5 promoter-pGL3 double restriction enzyme digestion; 2: mGluR5 promoter-pGL3 KpnI restriction enzyme digestion; 3: mGluR5 promoter-pGL3 HindIII restriction enzyme digestion. **c** Luciferase activity after treatment with various concentrations of pu-erh tea for 12 h. **d** Luciferase activity after treatment with 62.5 μg/mL pu-erh tea for various times. **e** Luciferase activity after treatment with 62.5 μg/mL pu-erh, black, green teas for various times. **f** Luciferase activity after treatment with 62.5 μg/mL pu-erh, black, green teas for 6 h. Data were analysed using the GraphPad Prism software (***p < 0.001 vs. vehicle control, *n* = 6)

To assess the role of pu-erh tea in transcriptional regulation and cell-specific expression of mGluR5, we constructed mGluR5 promoter-pGL3 recombinant plasmid The PCR products of the mGluR5 promoter were examined by 1 % agarose gel electrophoresis (Fig. 4a). PCR product is 1167-bp specific fragment consistent with the mGluR5 promoter. The promoter region was then subcloned into the *Kpn*I/*Hind*III sites of pGL3-Basic. Double enzyme digestion reaction yielded a clear obvious 1167-bp fragment, showing that a recombinant plasmid, mGluR5 promoter-pGL3 had been constructed (Fig. 4b).

Thus, the following detection on the activity of mGluR5 promoter to drive the expression of luciferase was assessed in HEK293T and SH-SY5Y cells. Luciferase activity driven by mGluR5 promoter was decreased significantly at a concentration of 62.5 μg/mL (Fig. 4c). The transfected cells were then incubated at this concentration for various times, with the result that at 6 h luciferase activity was decreased significantly (Fig. 4d). Compared to black and green teas, treatment with pu-erh tea for 6 h had a greater suppressive effect on transcriptional regulation of mGluR5 in both HEK293T and SH-SY5Y cells (Fig. 4e, f).

### Pu-erh Tea Inhibited the Expression of mGluR5

**Fig. 5:**
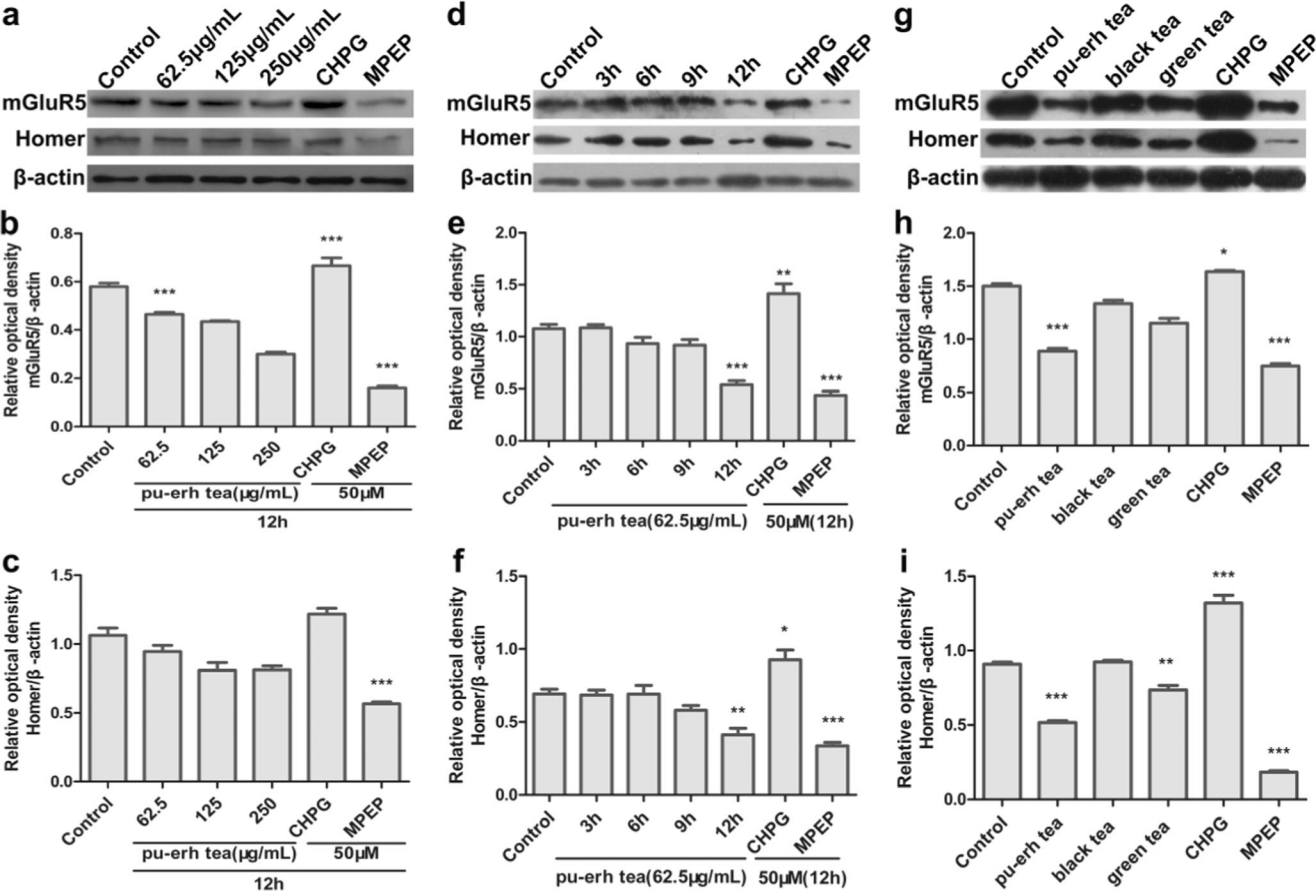
The expression of mGluR5 and Homer were detected by Western blot and analysed by ImageJ. **a**–**c** Cells were treated with various concentrations of pu-erh tea for 12 h. **d**–**f** Cells were treated with 62.5 μg/mL pu-erh tea for 3, 6, 9, 12 h. **g**–**i** Cells were treated with 62.5 μg/mL pu-erh, black, or green teas for 12 h. Data were analysed using GraphPad Prism software (***p < 0.001 vs. vehicle control, **p < 0.01 vs. vehicle control, *n* = 3)

To further validate the inhibition of the expression of the mGluR5, several concentrations of pu-erh tea, 50 μM CHPG (a selective mGluR5 agonist) and 50 μM MPEP (a selective mGluR5 antagonist) were added to SH-SY5Y cells and followed by incubation for 12 h. There were no effects on the growth of SH-SY5Y at the concentration of 62.5 μg/mL (Fig. 3c). The expression of mGluR5 was markedly reduced compared to the control when cells were treated with 62.5 μg/mL of pu-erh tea for 12 h compared to the control (Fig. 5a, b). When SH-SY5Y cells were treated with 62.5 μg/mL of pu-erh tea for various times, pu-erh tea markedly inhibited the expression of mGluR5 compared to the control (Fig. 5d, e). Compared to black and green teas, treatment with pu-erh tea for 12 h significantly inhibited the expression of mGluR5 (Fig. 5g, h).

The Homer family of proteins is localised predominantly at the postsynaptic density (PSD) in mammalian neurons and binds a specific proline-rich sequence on group I mGluRs. We assayed the expression of Homer by Western blot, and found that Homer was also significantly inhibited by pu-erh tea at dose-dependently and time-dependently (Fig. 5).

### Effects of Pu-erh Tea on Neuronal Injury

**Fig. 6:**
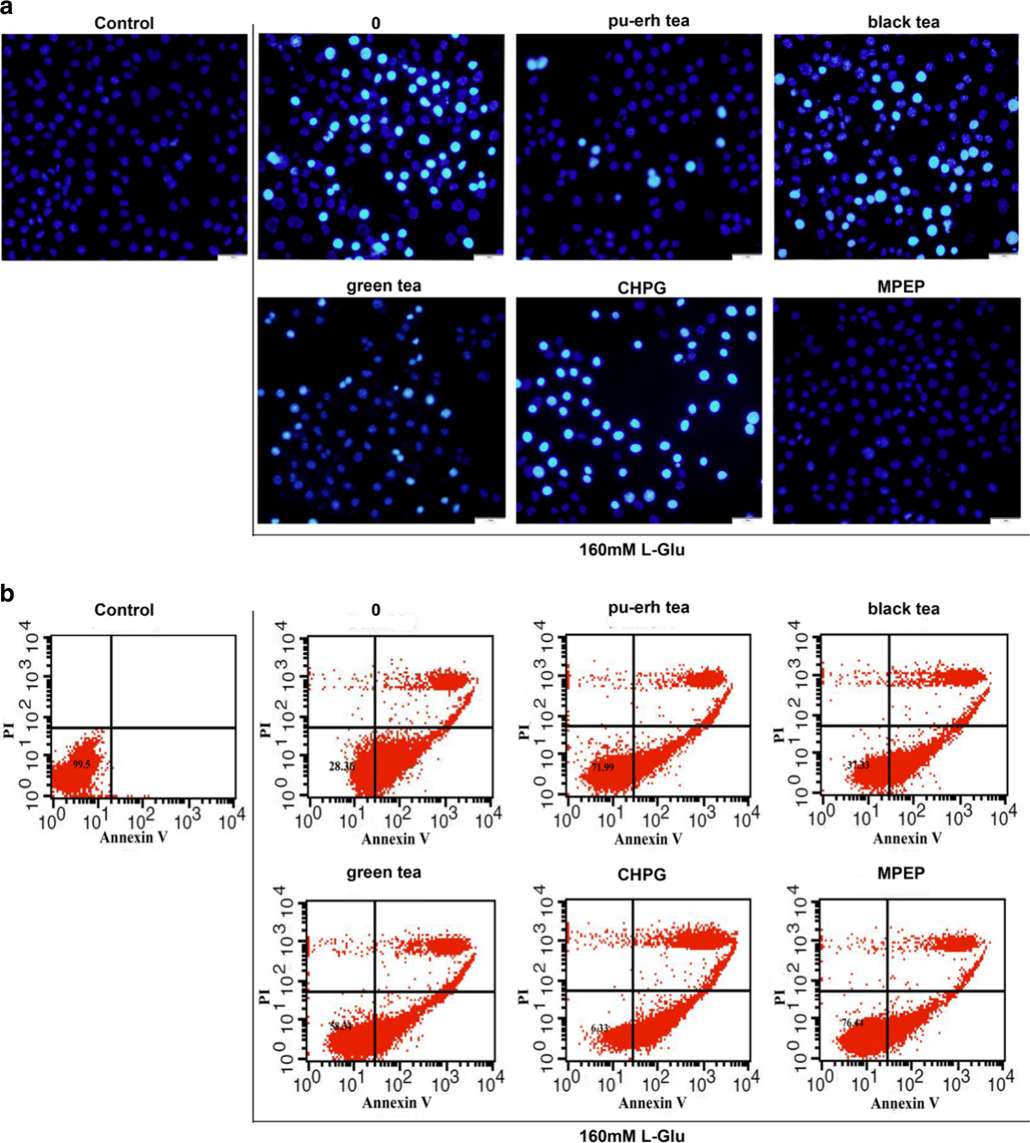

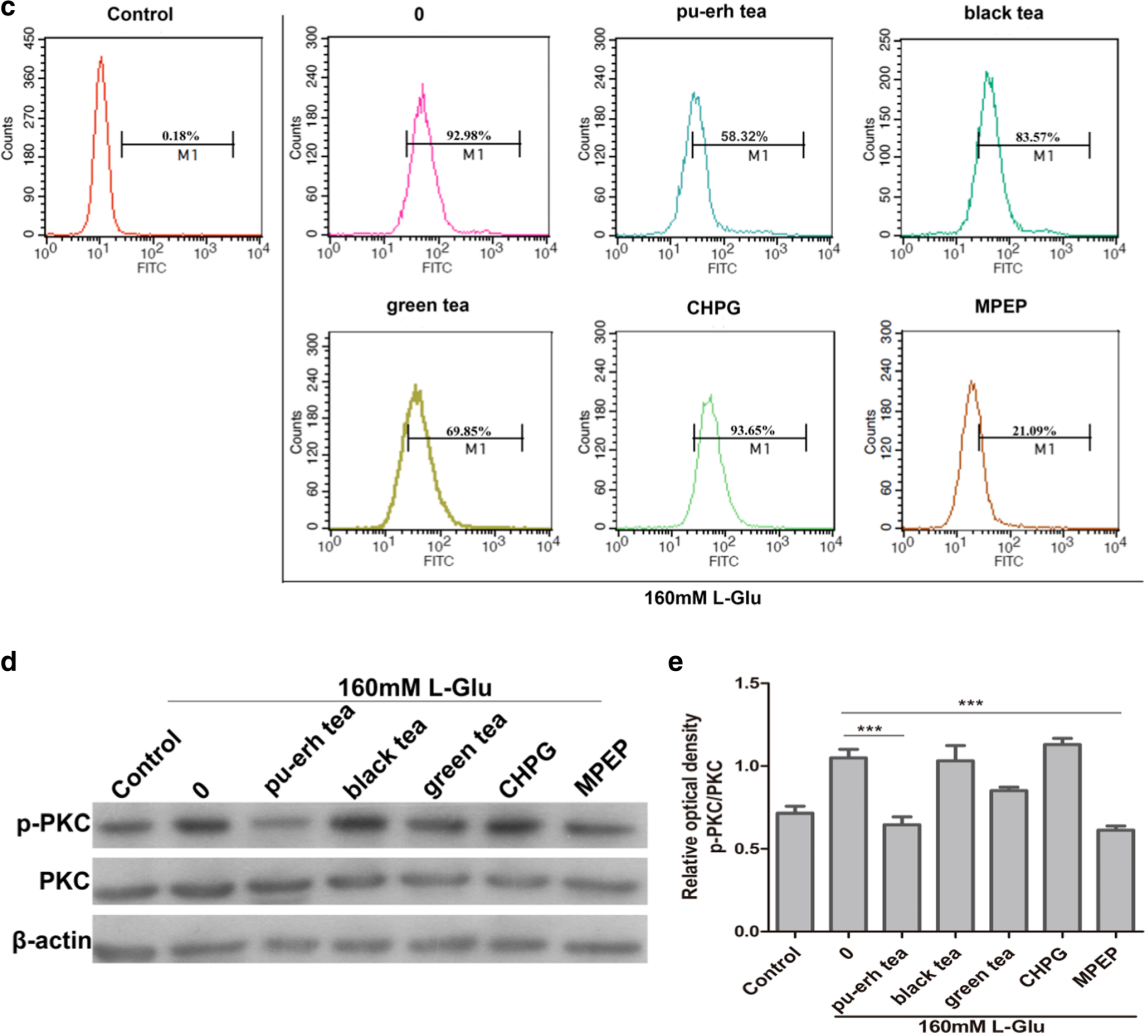
**a** Image of Hoechst 33342-stained cells. **b** Flow cytometry analysis of apoptosis. **c** Flow cytometry analysis of Ca^2+^ influx. **d** Western blot analysis of PKCα activity. **e** Quantification of protein level from three independent experiments using ImageJ. Cells were pretreated with 62.5 μg/mL pu-erh, black, or green teas for 12 h before treatment with 160 mM L-Glu. Data were analysed using GraphPad Prism software (***p < 0.001, *n* = 3)

Increase in extracellular levels of glutamate leads to activation of mGluR5 and a strong excitatory neurotoxicity, resulting in neuronal swelling and necrosis. To confirm that pu-erh tea protects cells from nerve injury, SH-SY5Y cells were incubated with pu-erh tea before treatment with L-glutamate (L-Glu). The nuclear morphology of SH-SY5Y cells was examined after staining with Hoechst 33342. Compared to black and green teas, 12-h treatment with 62.5 μg/mL of pu-erh tea reduced the number of hallmarks of apoptosis induced by 160 mM L-Glu, whereas the mGluR5 agonist CHPG increased and the mGluR5 antagonist MPEP decreased the number of apoptosis (Fig. 6a).

To further investigate whether pu-erh tea protects cells against apoptosis, the cells were analysed by flow cytometry after staining with Annexin V-FITC and PI. The percentage of surviving cells increased from 28.36 to 71.99 % after 12-h of treatment with pu-erh tea, whereas the mGluR5 agonist CHPG reduced the cell surviv to cells to 6.33 %. Treatment with black or green tea also increased the percentage of surviving cells (Fig. 6b).

### Involvement of Ca^2+^ in Prevention of Nerve Injury by Pu-erh Tea

The activation of mGluR5 could increase Ca^2+^ influx, and excitotoxic Ca^2+^ elevation is an early and possibly reversible pathological event that may lead to neuronal injury. To investigate the role of pu-erh tea in Ca^2+^ release in nerve injury induced by L-Glu, SH-SY5Y cells were incubated with pu-erh tea before treated with L-Glu, using an intracellular Ca^2+^ chelator (Fluo-3 AM). Pretreatment with pu-erh tea significantly inhibited calcium influx compared to that black tea or green teas. However, mGluR5 CHPG activated the calcium influx (Fig. 6c).

### Pu-erh Tea Inhibited PKCα Activation by L-Glu

In addition to Ca^2+^ release, PKCα is involved in mGluR5 signalling. After nerve injury by L-Glu, we observed that PKCα activity was increased. Moreover, pretreatment with the mGluR5 antagonist MPEP, inhibited PKCα activity. Pu-erh tea significantly reduced PKCα activity compared to black or green teas. The content of PKCα in each group did not change. (Fig. 6d, e).

### Effect of Pu-erh Tea on Behaviour

The Wistar rats that injected intraperitoneally with Licl-pilocarpine only had obvious epilepsy behaviour, while the groups treated with pu-erh tea onLicl-pilocarpine induced SE significantly attenuated the maximal seizure classes and the predominant behavioural seizure patterns in SE mice compared with the control (Table 3, *n* = 6).

**Table 3:** Effect of pu-erh tea on SE class

Pu-erh tea		−	−	−	+	+	+
LiCl-pilocarpine		−	+	+	−	+	+
Diazepam		−	−	+	−	−	+
Behaviour (times)	I/Class 1–2	0	32	5	0	8	4
	M/Class 3	0	51	1	0	5	0
	C/Class 4–6	0	64	0	0	0	0

### Pu-erh Tea Prevents Epilepsy by Inhibiting mGluR5

**Fig.7:**
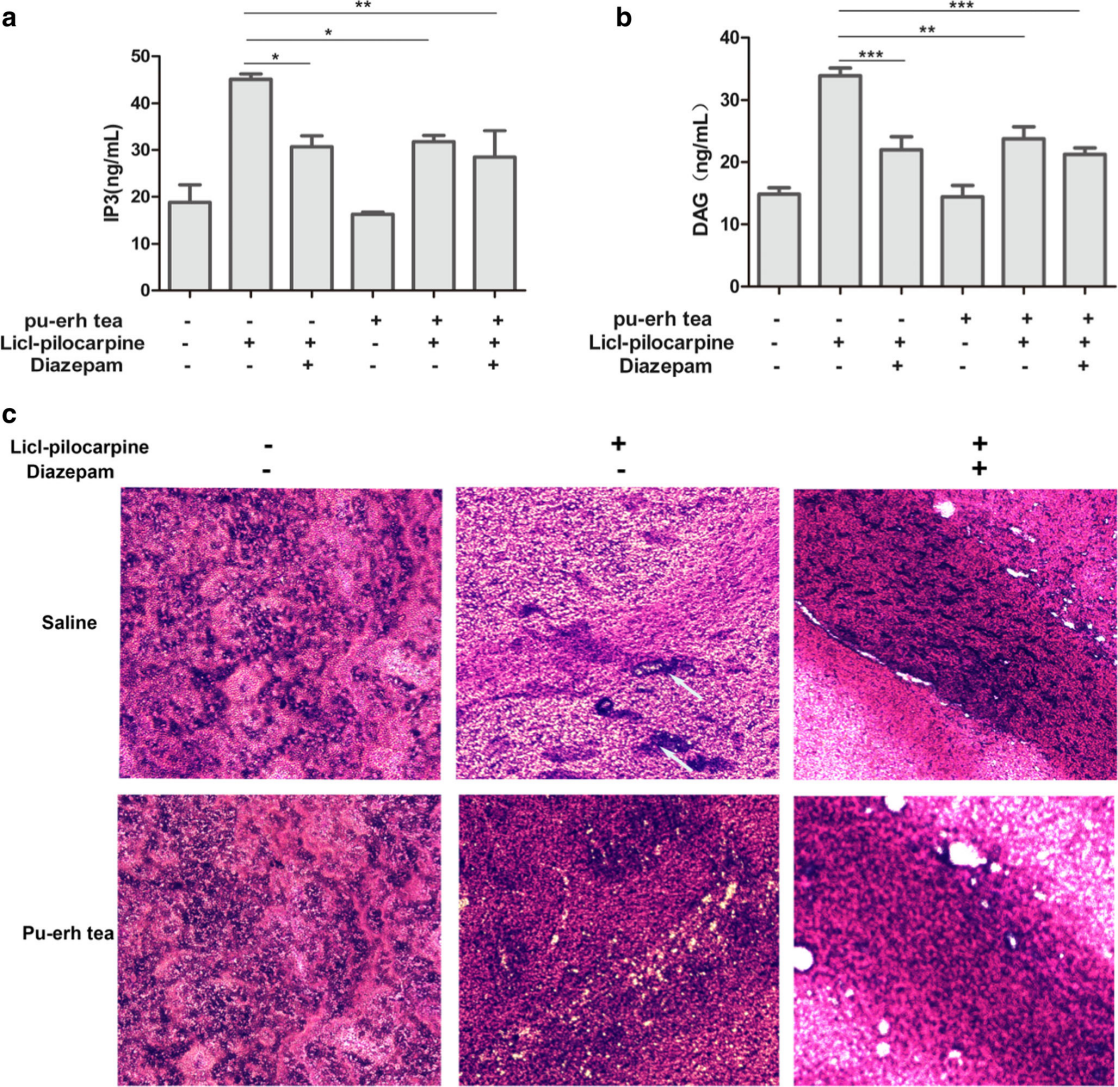

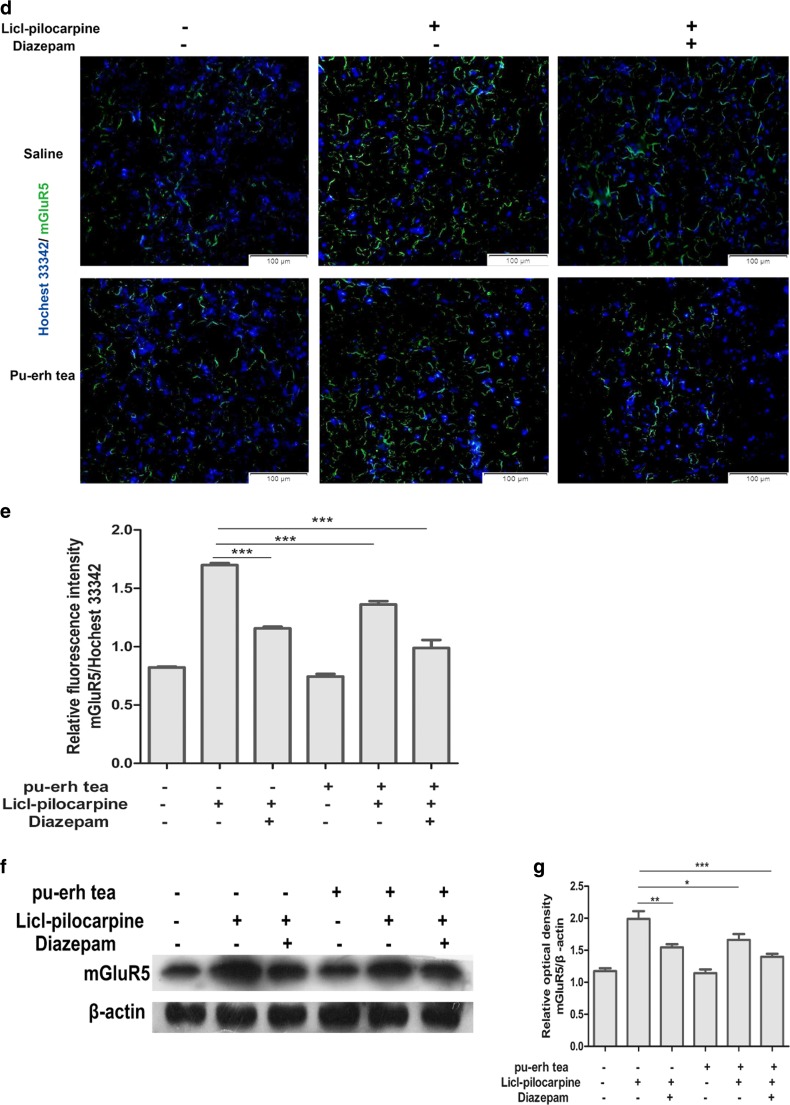
**a** Content of IP3 in the hippocampus. **b** Content of DAG in the hippocampus. **c** HE staining of hippocampus. **d** Immunofluorescence of hippocampus with Hochest 33,342 (blue) and anti-mGluR5 (green). **e** Quantification of relative fluorescence intensity of mGluR5/Hochest 33342 by ImageJ. **f** Western blot analysis the expression of mGluR5 in the hippocampus. **g** Quantification of protein levels from three independent experiments by ImageJ. Data were analysed using GraphPad Prism software (***p < 0.001, **p < 0.01, *p < 0.05, *n* = 3)

IP3 and DAG, as second messengers, play important roles on in intracellular messenger transduction. These two substances stimulate neurons and can cause neuronal damage if present in excess. We accordingly assayed the contents of IP3 and DAG in the hippocampus by ELISA to confirm the effect of pu-erh tea on prevention of epilepsy. The results showed that IP3 and DAG were increased in epilepsy and decreased under intragastric administration of pu-erh tea (Fig. 7a, b). Histological images using HE staining showed that after epilepsy rat hippocampus showed injury, whereas before intragastric administration of pu-erh tea or diazepam, rat hippocampus showed no changes compared to the control (Fig. 7c).

To determine whether mGluR5 is implicated in epilepsy, we used immunofluorescence and Western blot assays. In the immunofluorescence assays, the expression of mGluR5 was significantly upregulated in epileptic rats only administrated intraperitoneally with Licl-pilocarpin compared to others, whereas the rats before intragastric administration of pu-erh tea or diazepam showed reduced expression of mGluR5 compared to that in epilepsy (Fig. 7d, e). In Western blot, we also found the similar result (Fig. 7f, g).

## Discussion

In the present study, we observed that pu-erh tea decreased the mGluR5 promoter activity and downregulated the expression of the mGluR5 at dose-dependently and time-dependently. We also discovered that pu-erh tea protected SH-SY5Y cells against apoptosis induced by L-glu via blocking Ca^2+^ influx and inhibiting the PKCα activation. Pu-er tea had the role of alleviating epilepsy and reducing the expression of mGluR5 in epilepsy rats.

Human epidemiological and animal research experiments suggest that the pharmacological benefits of tea drinking may help protect the brain. And tea drinking has been shown to exert neuroprotective activities in a wide array of cellular and animal models of neurological disorders [25]. The Affymetrix expression microarrays showed that 0.25 mg/mL pu-erh tea decreased the mGluR5. Our results that pu-erh tea showed no toxicity to tumour cells and normal cells at low concentrations (≤ 0.25 mg/mL) was in accordance with the report that pu-erh tea showed no signs of toxicity and no deaths in SD rats at dosages ≤10 g/kg [26]. These results indicated that the reduction of mGluR5 mRNA and protein by pu-erh tea was not the result of cell death at low concentration (≤ 0.25 mg/mL).

Excessive mGluR5 activation has already been alluded to as a potential contributing factor in synaptic disorders, and a number of studies are currently testing the therapeutic potential of drugs that modify mGluR5 [27]. The modification of particular mGluR5 has been implicated in synaptic diseases and psychiatric disorders [28]. Despite progress in drug treatment of schizophrenia, cognitive and other negative symptoms remain a major issue of the disease, often representing residual symptoms of resistant schizophrenia and being worsened by the side effects of antipsychotics effects [29].

The mGluR5 promoter we cloned could regulate the expression of luciferase (Fig. 4). It was consistent with the result that the alternative 5-splicing and usage of multiple promoters may contribute regulatory mechanisms for tissue- and context-specific expression of the mGluR5 gene [30]. The results of luciferase assays (Fig. 4) and Western blot (Fig. 5) suggested that pu-erh tea contains active compounds that could inhibit the transcription and the translation of mGluR5 compared to black tea and green tea. It may be during the fermentation, the types, activity and content of the components in pu-erh tea are different from those of black and green teas [31]. Owing to the complexity of the composition of pu-erh tea, the active ingredients that protect neural cells via reducing the expression of mGluR5 await further study. The mGluR5 agonist CHPG increased the expression of mGluR5 and an mGluR5 antagonist MPEP reduced the expression of mGluR5 (Fig. 5). These results suggested that pu-erh tea was a candidate as mGluR5 antagonist.

Importantly, the activity of mGluR5 is tightly regulated by its association with scaffolding protein, Homer [32]. In Western blot, we found that pu-erh tea could downregulate the expression of Homer significantly compared to black or green teas (Fig. 5). Homer proteins share a commom Ena/Vasp homology protein (EVH1) domain at the N-terminus that binds to the intracellular C-terminal tail of mGluR5 and localises mGluR5 to the postsynaptic density (PSD) [6, 33, 34]. In addition, it has been shown that disruption of mGluR5-Homer interactions selectively blocks mGluR5 activation [35]. These results indicated that the inhibition of mGluR5 by pu-erh tea disrupted mGluR5–Homer scaffolds, resulting in decreasing Homer.

A recent study suggests that enhanced mGluR5-Homer binding leads to a reduction in mGluR5-induced cytosolic Ca^2+^ increase [36]. However, in our study, both L-Glu and mGluR5 agonist CHPG induced nerve cell necrosis and increased intracellular Ca^2+^ response. These findings are in agreement with the report that Homer proteins bind IP3 receptor [37]. IP3 induces calcium release from intracellular stores, potentially leading to cell death [38]. Pretreatment with pu-erh tea or an mGluR5 antagonist MPEP could protect cells from necrosis and decrease intracellular Ca^2+^ response. These results suggested that pu-erh tea had the role of mGluR5 antagonist that prevented Homer binding IP3 receptor, which contributed to blocking the glutamate stimulation and inhibiting the release of intracellular Ca^2+^ in response to neuronal injury.

The mGluR5-induced Ca^2+^ response could also be negatively regulated by Homer 1a under some condition [39]. Given that group I mGluRs are positively coupled to PLC, leading to PKC activation and phosphorylation of serine 839 mediated by PKC activation is involved in the regulation of mGlu5 receptor-mediated Ca^2+^ influx [40, 41]. Therefore, we wondered whether the ability of pu-erh tea to attenuate the release of intracellular was dependent mGluR5-PKC. We tested the activation of PKCα and found that both L-Glu and the mGluR5 agonist CHPG stimulated PKC activation, whereas pu-erh tea or an mGluR5 antagonist MPEP inhibited PKCα activation. Thus, we theorise that pu-erh tea protected neuronal injury by inhibiting PKC activation which resulted from inhibiting the release of intracellular Ca^2+^. However, PKC isoenzymes have been classified into two subfamilies: the conventional subtypes (cPKC: α, βI, βII, γ) are Ca^2+^ and DAG-dependent and novel subtypes (nPKC: δ, γ, η, θ,) are DAG-dependent, but Ca^2+^-independent [42]. Group I mGluRs coupled to Gαq/11 proteins stimulate the activation of PLC, resulting in DAG formation. Pu-erh tea decreased the contents of IP3 and DAG in the hippocampus (Fig. 7a, b). Accordingly, whether pu-erh tea influences the novel subtypes of PKC awaits further investigation.

**Fig. 8:**
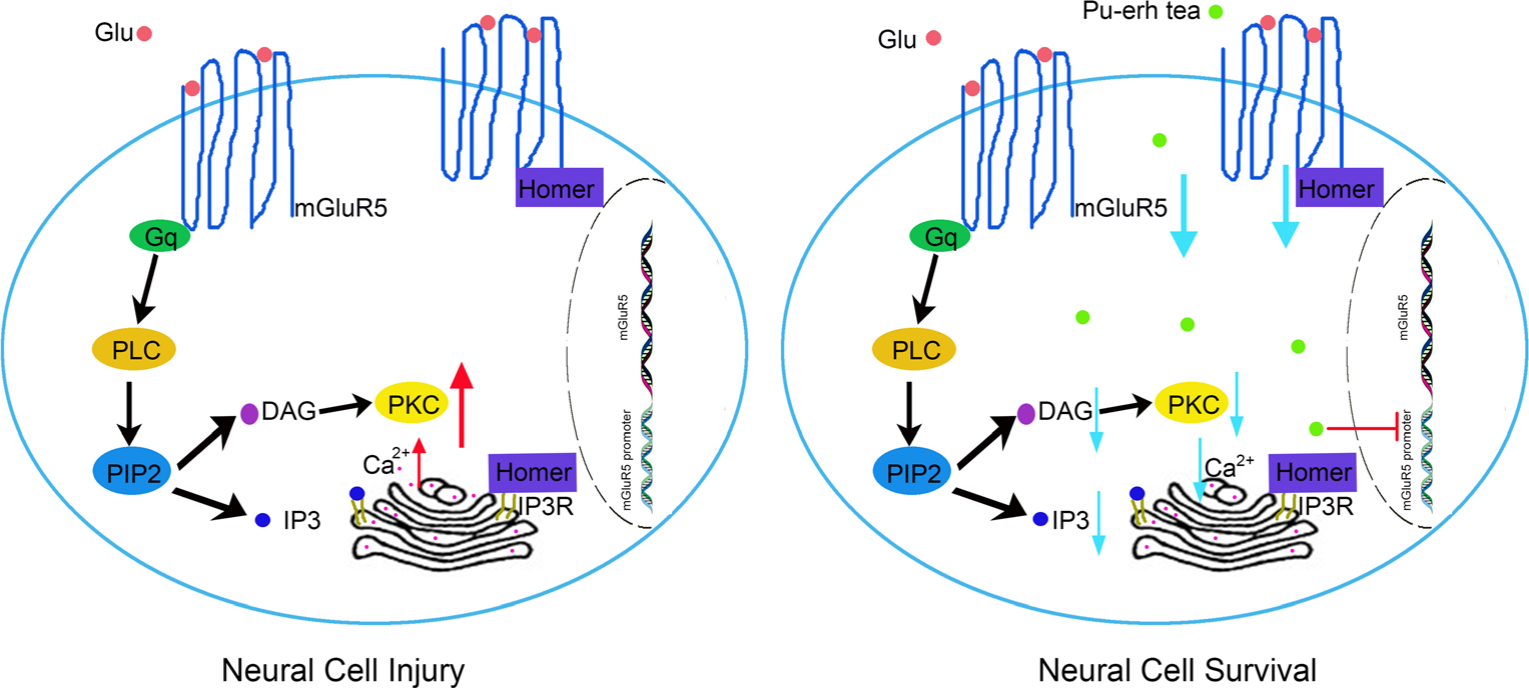
The mechanism of pu-erh tea protects neural cell from injury stimulated by glutamate

mGluR5 is well known to play important roles in neuronal function and synaptic plasticity, and defects in mGluR5 signals are thought to cause a variety of neurological disorders. Activation of mGluR5 increases epileptiform activity and an mGluR5 antagonist MPEP inhibits convulsive and non-convulsive primary generalised seizures [43]. In this study, we constructed an epilepsy model by using Licl-pilocarpin [21]. Pilocarpine activates the G-protein which mediates the receptor stimulation to PLC. PLC hydrolyses PI into IP3 and DAG. IP3 and DAG cause neuronal stimulation and when in excess, may contribute to neuronal injury [44]. In general, mGluR5 combined with glutamate activates the G-protein Gq/11, and subsequently, the activated G-protein activates PLC which hydrolyses membrane phosphoinositides to form IP3 and DAG [5]. We found that pu-erh tea could decrease the severity of seizure behaviours and the content of IP3 and DAG. Immunofluorescence and Western blot both showed that pu-erh tea reduced the expression of mGluR5 that was increased in epilepsy rats. These results coincide with that the expression of mGluR5 is higher in the hippocampus of epileptic patients, than in those of a control group [45]. The results indicated that pu-erh tea could decrease the content of IP3 and DAG through reducing the expression of mGluR5. Taken together, these results indicate that pu-erh tea played an important role in protecting neural cells from injury induced by glutamate and antiepileptic via inhibiting the activity of mGluR5 promoter and the expression of mGluR5 (Fig. 8).

## Conclusion

Dysregulation of mGluR5 is implicated in multiple brain disorders. Accordingly, many selective mGluR5 antagonists have been developed for clinical therapy. However, these drugs have produced unacceptable side effects in clinical trials. The findings of this study suggested that pu-erh tea can inhibit mGluR5 activation stimulated by glutamate via inhibiting the activity of mGluR5 promoter and the expression of mGluR5, which blocks Ca^2+^ influx and suppresses the activity of PKC α to protect nerve cell from necrosis. Pu-erh tea also relieves rat epilepsy and reduce the contents of IP3 and DAG in epilepsy rats. These results suggest that pu-erh tea is a potential neuroprotective agent acting via regulation of mGluR5. However, the specific compounds in pu-erh tea that function in neural protection await further study.
